# ZnSe-rGO nanocomposites as photocatalysts for purification of textile dye contaminated water: A green approach to use wastewater for maize cultivation

**DOI:** 10.1016/j.heliyon.2023.e22687

**Published:** 2023-11-23

**Authors:** Mishal Zahra, Ghazala Yasmeen, Faryal Aftab, Habib-ur-Rehman Athar, Aisha Saleem, Sarah Ambreen, Muhammad Aslam Malana

**Affiliations:** a Physical Research Laboratory, Institute of Chemical Sciences, Bahauddin Zakriya University Multan, Punjab, Pakistan; b Department of Chemistry, The Women University Multan, Punjab, Pakistan; c Institute of Botany, Bahauddin Zakariya University, Multan, 60800, Pakistan

**Keywords:** ZnSe-XrGO nanocomposite, Photodegradation, Methyl violet, Seeds germination, Plant growth

## Abstract

Disputes about the probable availability of safe water and the efficacy of processed wastewater are key issues that necessitate a suitable solution to enhance the quality of clean water. The current research emphasizes the synthesis of ZnSe-reduced graphene oxide nanocomposites (ZnSe:rGO) with different weight ratios of rGO (represented as X = 0.6, 1 and 1.6 g)via one-step hydrothermal method. The photocatalytic performance for the degradation of methyl violet (MV) dye was investigated under visible light irradiation by varying the reaction parameters. The crystal structure, elemental composition, surface functionality and morphology of the synthesized ZnSe-XrGO nanocomposites were estimated by powder X-ray diffraction (XRD), energy dispersive X-ray spectroscopy (EDX), Fourier transform infrared spectroscopy (FTIR) and scanning electron microscopic (SEM) techniques. UV–visible spectroscopy was used to investigate the optical properties. The highest efficiency is obtained for ZnSe-XrGO in 1:1 and it showed pseudo 1st order behavior with rate constant of 0.0167min^−1^and 94 % photodegradation of MV in just 3 h. Furthermore, hazardous effects of MV were investigated on the germination and growth of *Zea mays* seeds by giving them aqueous solution of MV (0, 8, 12, 24 and 48 ppm) and the decontaminated water after photodegradation of MV with the synthesized photoactive composite. The results showed profound negative effect on both germination and seedling growth at higher concentration (>12 ppm) of the dye solution. No hazardous effects were observed on both these parameters when it was given the dye degraded water which reflects the practical use of the synthesized catalyst for water remediation. The current study fulfills the goal of designing an efficient visible-light active nano-photocatalyst and its direct applicability on life sciences for water purification.

## Highlights

• ZnSe nanoparticles supported over graphene sheets synthesized via one-step hydrothermal method.
• Different composite nanostructures were prepared by varying amount of rGO and the ZnSe-XrGO nanocomposite with x = 1 showed excellent performance in photodegradation of methyl violet (MV).
• The seed germination and growth of maize (*Zeamays* L.) plants was greatly affected when supplied with dye-contaminate water upto 24–48 ppm.
• Irrigation with degraded-dye water (purified) by the synthesized composite had minimal adverse impact on seed germination and growth of maize plants.

## Graphical abstract

**Figure:**
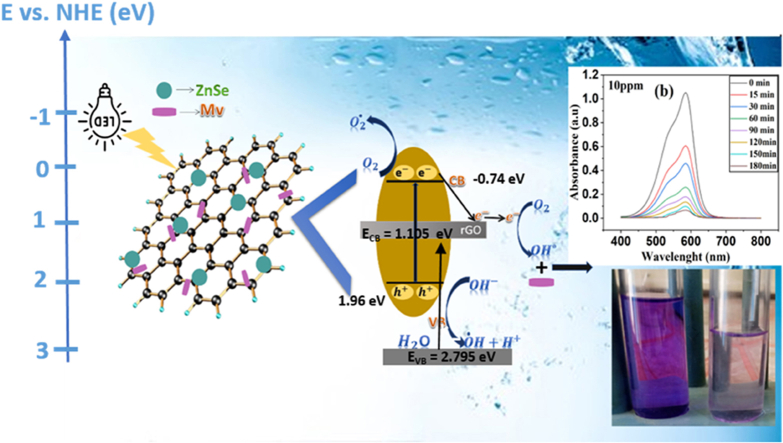

## 1 Introduction

Water is a necessary component of life. Water contamination from numerous contaminants, such as pesticides, dyes, pharmaceuticals, heavy metals, cosmetics, and so on has worsened the availability of purified water [1]. The uncontrolled release of wastewater into the environment from hospitals, sewage, industries, and other sources is the primary cause of water pollution. Among different contaminants, water pollution due to dyes coming from the discharge of textile industries, is of particular interest, prompting scientists to develop innovative ways for water treatment [2,3]. According to reports, around 20 % of the dyes are drained into the ecosystem's water as industrial effluent [4]. Increased dye accumulation threatens aquatic life and the natural environment by interfering with nature's equilibrium [5]. Consequently, the existence of dyes and their derivatives in wastewater is a worldwide issue that must be addressed immediately. Because of their stable structures, these dyes are insensitive to biological oxidation and harmful to both the plant and animal kingdoms [6]. Furthermore, if dyes are accumulated in large values, they can cause serious problems such as skin infections, genetic mutations, liver damage, and allergies [7].

Methyl violet (MV), a brightly colored triphenylmethane cationic dye, is additionally referred to as Basic violet-I, used extensively in different industries like leather, textile, tonners, ink, printing, rubber and plastic etc. for coloring purpose [8]. This dye, on the other hand is a refectory dye which may stay in the environment for a long period and cause harm [9]. MV has been identified as mutagenic and carcinogenic dye, additionally it's an irritant to the skin, eyes, respiratory, and gastrointestinal systems [10]. It also inhibits microbial growth and affects photosynthetic responses thus affecting both plants and animals. To address these concerns, such as low-concentration pollutant discharge into water directly, our planet requires immediate action to protect the environment from sources of pollution [11,12]. For the removal of dye contaminants from wastewater, several technologies and approaches have been investigated. These include adsorption [13], precipitation [14], reverse osmosis [15], ion exchange [16], and other typical water treatment processes. However, traditional methods have their own drawbacks, including high operating expenses, and increased energy consumption [13]. These all techniques just extract the dye from water but its hazardous effect still remains the same as the dye is still intact. Apart from these traditional techniques, biological processes using microorganisms (such as algae, fungi, and bacteria) for dye removal have also been investigated. The advanced oxidation process (AOP) is a straightforward method for producing aggressive oxygen species like superoxide radical and hydroxyl radical for initiating the oxidation of organic pollutants [17].

Heterogeneous photocatalysis is an advanced oxidation process (AOP) that takes place on the photocatalyst's surface and is an efficient method for removing and degrading organic pollutants from a water stream. The biggest advantage is that the photocatalyst generates the active oxygen species by utilizing sunlight, which may further react with the carcinogenic dye and convert it into harmless species. Thus, the process makes the water ecofriendly by completely eliminating the hazardous material from environment. A photocatalyst is a semiconducting material which is stimulated by photon energy equivalent or more than the band-gap energy (λ≥ Eg) of the material, resulting in the formation of photogenerated electron-hole pairs (EHP). For improved photocatalytic efficiency of semiconductors, the separation of photo induced holes and electrons is necessary [18]. These photogenerated EHP react with absorbed water and oxygen molecules, consequently O_2_°^-^ and ^◦^OH radicals are formed [19]. The simultaneous assault of these radicals on long chain conjugation of organic dye permits the degradation of toxic organic pollutants into environment friendly species, such as water and carbon dioxide [20].

Nano-sized semiconductors have received increased attention due to their greater surface area, size-dependent physiochemical features, and strong photocatalytic activity, which make them a potential candidate for wastewater treatment. Nanomaterials based on TiO_2_ are extensively developed and most explored for photocatalysis due to their extraordinary photocatalytic activity (because of its wide band gap (3.2eV), strong oxidation capability, abundance, cost effectiveness and harmless effects on environment. But this wide gap limits its efficiency as it utilizes only high energy UV region of solar spectrum (4 % of sunlight) for the generation of EHP species [21]. On the other hand, many transition metal chalcogenides (e.g. Se, Te and S) have demonstrated significant photocatalytic activities in recent years and have remained a hot research topic. Among these ZnSe nanoparticles showed outstanding band-gap of **∼**2.7eV and large exciton binding energy of **∼**20 meV for photodegradation. Recently, Qian et al. (2007) have revealed that ZnSe has better efficiency than TiO_2_ for photodegradation of magenta acid dye, under ultraviolet (UV) irradiation [22]. ZnSe is being extensively studied as photocatalyst by various other researchers because of low cost, high stability visible light activity, and less toxicity. Zang et al. (2021) reported spherical shaped flower-like structure of ZnSe which showed better photocatalytic efficiency under sunlight irradiation for the degradation of methyl orange and showed 96 % degradation in 9 h [23,24]. However, the photocatalytic efficiency is suppressed by rapid rate of EHP recombination in ZnSe making the process more time consumable and economically less feasible. Different ways (e.g. developing heterojunctions [25], doping of non-metals N, P [26]and loading over carbonaceous support [27] have been used to separate the photogenerated electron-hole pairs and supporting the nanoparticles over some carbon based material of high conductivity is a promising strategy [28].

Graphene and its derivatives having a two-dimensional honeycomb structure is thought to be a promising optoelectronic material, owing to their remarkable electric and photonic properties with excellent electron mobility, flexible structure, large specific surface area, high transparency and stability at room temperature. An extraordinary property of graphene is that it has ability to absorb photon across a wide spectrum, from infrared to visible region [29,30]. Various reports showed that graphene-based semiconductor (more specifically ZnSe) nanocomposites suppress the EHP recombination and enhance the transfer of photogenerated-charges resulting in increased photocatalytic efficiency in comparison to individual entities [29]. For example, S.H. Hsieh et al. (2015) reported ZnSe/Graphene for the photodegradation of methylene blue, Which showed high photocatalytic efficiency (99.6 % in 6 h s under visible light) than ZnSe [29].P. Chen et al. (2012) used a catalyst-free technique to synthesize nitrogen-doped graphene/ZnSe (GN-ZnSe) composites which were proved to be efficient for the photodegradation of Methyl orange (MO). In comparison to commercial ZnSe (only 7.84 % MO was removed), 30.72 % MO was ingested in 8 hs over GN-ZnSe composite [31]. Liu et al. (2013) synthesized ZnSe nitrogen doped graphene (ZnSe/N-Gs) and ZnSe.xN_2_H_4_/graphene(ZnSe.N_2_H_4_/GS) nanocomposites via one-pot solvothermal method. The photocatalytic activity of the nanocomposites was investigated by the photodegradation of methylene blue dye under visible light and photocatalytic hydrogen production via water splitting. The degradation efficacy of MB while using (ZnSe.N_2_H_4_/GS) was 38.2 % in 4.5 h s but (ZnSe/N-Gs) removed methylene blue almost 80 % [32]. But in the available reports the dye degradation efficiency was not really good and the process takes much time that is not economically feasible and most of the available reports are on the MB and MO dye only. To the best of our knowledge there is no report available on the one pot synthesis of ZnSe-rGO nanocomposite and its photocatalytic degradation ability for MV dye. Also, uptill now, no one has scaled up the experiment to test the direct impact of dye degraded water on life sciences.

Here, we are reporting one-step hydrothermal synthesis of ZnSe nanoparticles decorated on the sheets of reduced graphene oxide nanocomposites(ZnSe-rGO). We investigated the effect of ZnSe-rGO ratio in nanocomposite and reaction conditions on the photocatalytic degradation efficiency of MV. The use of cured water from dye (after its degradation from the synthesized nanocomposite) for germinating and growing Zea Mays seeds is the novelty of this investigation.

## 2 Experimental details

### 2.1 Materials

Graphite powder(C), and Zinc chloride(ZnCl_2_) were purchased from DAEJUNG, selenium powder(Se) from CICA BRAND, Hydrazine monohydrate(N_2_H_4_–H_2_O), potassium hydroxide(KOH) and phosphoric Acid(H_3_PO_4_) from DUKSAN, Sulphuric acid(H_2_SO_4_) from ANALAR, Hydrogen peroxide(H_2_O_2_) and methyl violet(MV) from E. MERK. These entire chemicals were utilized without any further purification.

### 2.2 Synthesis of ZnSe-rGO nanocomposites

**Fig. 1:**
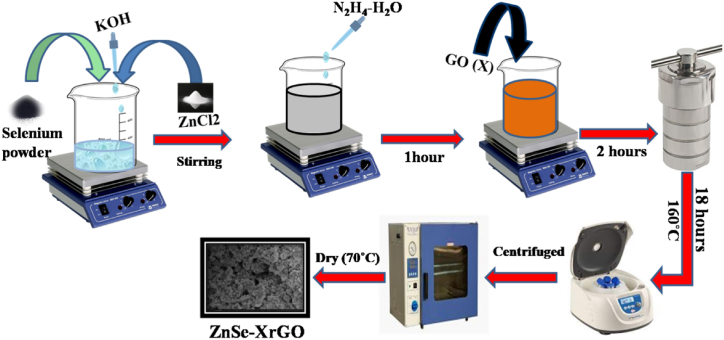
Fabrication Process of ZnSe-XrGO nanocomposites.

Graphene oxide(GO**)** was synthesized by the modified hummer's method by the oxidation of graphene [27,33]. ZnSe-rGO nanocomposite was synthesized by hydrothermal method (Fig. 1). It is one of the best methods for the preparation of nanoparticles because of simpler setup and involvement of mild conditions. In this process, 0.47 g Se powder was dispersed in 60 mL of the deionized water, 0.8 g ZnCl_2_ and 6 M KOH solution were added to it. After 5 min, 10 mL hydrazine monohydrate was added to above mixture which was stirred for 1 h at ambient temperature. The required amount of graphene oxide (GO) was added and stirred for 2 h again. Following that, 80 mL of obtained mixture was transferred to the 100 mL Teflon lined-stainless steel autoclave at 160 °C for 18 h. The obtained precipitates were collected by centrifugation and rinsed repeatedly by ethanol and deionized water. The synthesized ZnSe-rGO precipitates were dried in oven at 70 °C for overnight. The obtained nanocomposite was denoted as ZnSe-XrGO where X represents the different weight ratios of rGO. The ratio ZnSe to rGO was (1:0.6, 1:1, 1:1.6) and are represented here as ZG1, ZG2 and ZG3, respectively.

### 2.3 Characterization

Surficial morphology, size distribution, elemental composition and chemical purity of the ZnSe-XrGO nanocomposites were investigated by scanning electron microscopy(SEM, Nova Nano SEM 450) and electron X-ray diffraction (EDX). The crystal structure, X-ray dislocation density, d-spacing and average crystallite size were evaluated by using powder X-ray diffraction (XRD, Bruker D2Phaser XRD) at 10–80° with Cu-Kα radiations. The functional groups identification within the synthesized nanocomposites was done by Fourier transform infrared spectroscopy (FTIR, Alpha FTIR spectrometer, Bruker). The optical characteristics(photocatalytic efficiency) were examined by using UV-VIS spectroscopy (Shimadzu UV-1800) and band gap was measured via Agilent Carry 60 UV–Visible doublebeam spectrophotometer. The chlorophyll content in maize plant was measured by using chlorophyll meter (SPAD-502, Sensing, Konica Minolta, Inc., Japan) and chlorophyll fluorescence meter (PAR-FluorPen FP-100, PSI, Czech Republic).

### 2.4 Photodegradation of MV

Aqueous solution of MV (10 ppm) was prepared and photocatalyst ZnSe-XrGO(10 mg) was added. The test solution was magnetically stirred under dark for 0.5 h to obtain adsorption-desorption equilibrium. To start photoreaction, solution was exposed to visible light (Commercial incandescent tungsten bulb, 200 W) for 3 h. With the help of syringe, 3 mL of the solution was taken out of the photoreactor and centrifuged to remove the photocatalyst. The concentration of MV was measured by recording absorption of the supernatant using UV-VIS spectrophotometer (Shimadzu UV-1800). The degradation of methyl violet was investigated by tracking the decrease in major absorption peak of methyl violet. The %age degradation and kinetics of degradation were calculated by the following equations (Eq. (1)):

(1) $$Degradation\;effeciency\left(\%\right)=\left(1-\frac{C}{C_{o}}\right)\times 100$$

where C₀ and C are the concentrations of methyl violet in solution at time 0 and *t* min, respectively [29,30]. The photodegradation rate constant was calculated by the equation given below as (Eq. (2)) [34]:

(2) $$\ln\left(\frac{C₀}{C}\right)=kt$$

where, k (min^−1^) is the degradation rate constant.

### 2.5 Effect of dye contamination on seedling growth and germination of plant of maize (*Zeamays* L*.)*

To investigate the effect of dye contamination on seedling growth and germination of*Zea mays* plant, aqueous solution of different concentrations of methyl violet dye (0.0, 8.0, 12, 24 and 48 ppm) were prepared and stored in appropriately labeled measuring flasks. The Treatment levels prepared were: 0.0 ppm solution (control, C1), degraded dye solution (D1), 8.0 ppm dye solution (T1), 12 ppm dye solution (T2), 24 ppm dye solution (T3) and 48 ppm dye solution (T4).

In order to evaluate stress due to dye contaminated water on maize plants, seeds of maize were treated with various grades of dye solutions. The effect of the contaminants on germination and seedling growth was monitored for a period of 10 days. Seeds of maize (var. Almas) obtained from local seed market. Seeds of maize were surface sterilized 5 % NaOCl and washed with distilled water thrice. Twenty seeds of maize were placed in 18 cm diameter Petri dishes that were double lined with filter paper (Whatman filter paper 2) and moistened with 5 mL of different concentrations of MV dye solution (0, 8, 12, 24 and 48 ppm dye; and photodegraded dye). Each treatment was replicated thrice. Evaporative water loss from Petri dishes was compensated by adding distilled water and thus maintained the volume of applied solution. Whole set of experiment was conducted in growth room with PAR 300 μmol m^−2^ s^−1^, temperature 25 °C ± 3; relative humidity 50–65 %. Seed germination percentage was recorded daily over a period of 10 days. After which seedlings were carefully uprooted and separated into shoots and roots. Data for lengths of shoots and roots, fresh weights of shoots and roots were recorded. Seedling parts were then oven-dried at 70 °C for 72 h and dry weights were measured.

Before the harvest, leaf chlorophyll contents were recorded using chlorophyll content meter (SPAD-502, Minolta, Japan). In addition, plant photosynthetic capacity was assessed using chlorophyll fluorescence meter (PAR-FluorPen FP-100, PSI, Czech Republic). Before the taking measurements, leaves were dark adapted by wrapping leaves with aluminum foil for 30 min. A weak measuring light (0.01 μmol m^−2^ s^−1^) was applied to measure Fo (minimal fluorescence) and then a strong saturated pulse of 3000 μmol m^−2^ s^−1^ for 0.6 s was applied to measure Fm (maximal fluorescence). Quantum yield of photosystem II (PSII) was calculated as Fm – Fo/Fm.

The obtained data was subjected to One-Way analysis of variance (ANOVA) using computer package CoStat 6.6 (Cohort, Berkeley, USA). Means were compared with least significant difference (LSD) at 5 % probability level.

## 3 Result and discussion

### 3.1 Structural analysis

**Fig. 2:**
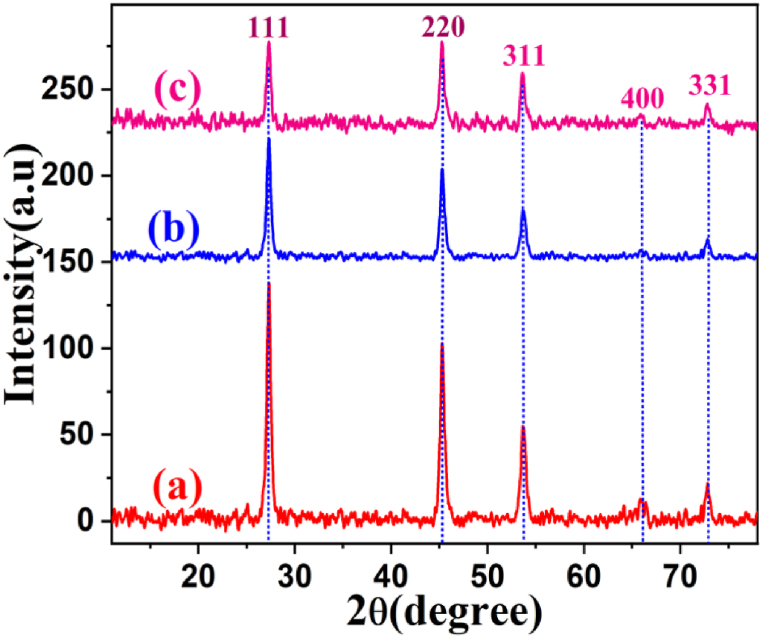
XRD spectra of (a) ZG1 (b) ZG2 (c) ZG3 nanocomposites.

Structural description and phase purity of the synthesized material was assessed by powder X-ray diffraction (XRD) technique. The diffraction patterns of ZG1, ZG2 and ZG3 are compared in Fig. 2. The XRD analysis of nanocomposites at different light intensities showed the pattern of narrow peaks indicating high crystallinity. The crystalline structure was evaluated by comparing the pattern with standard [Ref No.00-005-0522] and the patterns showed peaks at diffraction angles (2θ) of 27.3°, 45.31°, 53.6°, 66.3° and 72.74° corresponding to (111), (220), (311), (400) and (331) *hkl* lattice planes respectively. This confirms all the characteristic peaks of cubic phased ZnSe in the tested sample and no obvious peak for graphene which might be due to the mixing with the peak of ZnSe (111) plane that appears very close to (002) plane of graphene [35,36]. The patterns match exactly with the standard card [Ref No.00-005-0522] and no obvious impurity phase like ZnO is observed, indicating high purity of product [37] Average crystallite sizes of ZG1, ZG2 and ZG3 are 19.6 nm, 21.9 nm and 19.6 nm respectively calculated by Debye Scherrer equation (Eq. (3)) [38]:

(3) $$Crystallite\;size\left(D\right)=\frac{K\lambda }{\beta \;\mathrm{Cos}\;\theta }$$

where K is Scherrer's constant and refers to shape of the crystal, β is the full width at half maxima (FWHM), λ is X-ray's wavelength, θ is the Bragg's angle, and D is crystallite size [39]. X-ray data profile have also been utilized to calculate dislocation density (ẟ) from equation (4) [40]:

(4) $$ẟ=\frac{1}{D^{2}}$$

The dislocation density is related with the surface roughness of the material which affects the photocatalytic efficiency of semiconductor [41,42]. Furthermore, as the dislocation density decreases, the crystallinity of the material also increases [43]. The dislocation density of the ZG2 (2.2 × 10^15^ m^−2^) was competitively lowest among the synthesized nanocomposites which result in higher crystallinity making the photocatalyst more efficient.

The crystallinity (%) was also estimated by using equation (5), it was 51 %, 57.2 % and 49 % for ZG1, ZG2 and ZG3 nanocomposites, respectively. These results are in line with the above argument from dislocation density and ZG2 showed the maximum crystallinity among all the samples, making it more effective for photocatalytic process [43,44].

(5) $$Crystallinity\left(\%\right)=\frac{Area\;under\;crystalline\;peaks\;\left(A_{CP}\right)}{Area\;of\;all\;the\;peaks\;\left(A_{AP}\right)}\times 100$$

$$\left(A_{AP}=Area\;of\;Crystalline\;peaks+Area\;of\;Amorphous\;peaks\right)$$

### 3.2 Surface functionality

**Fig. 3:**
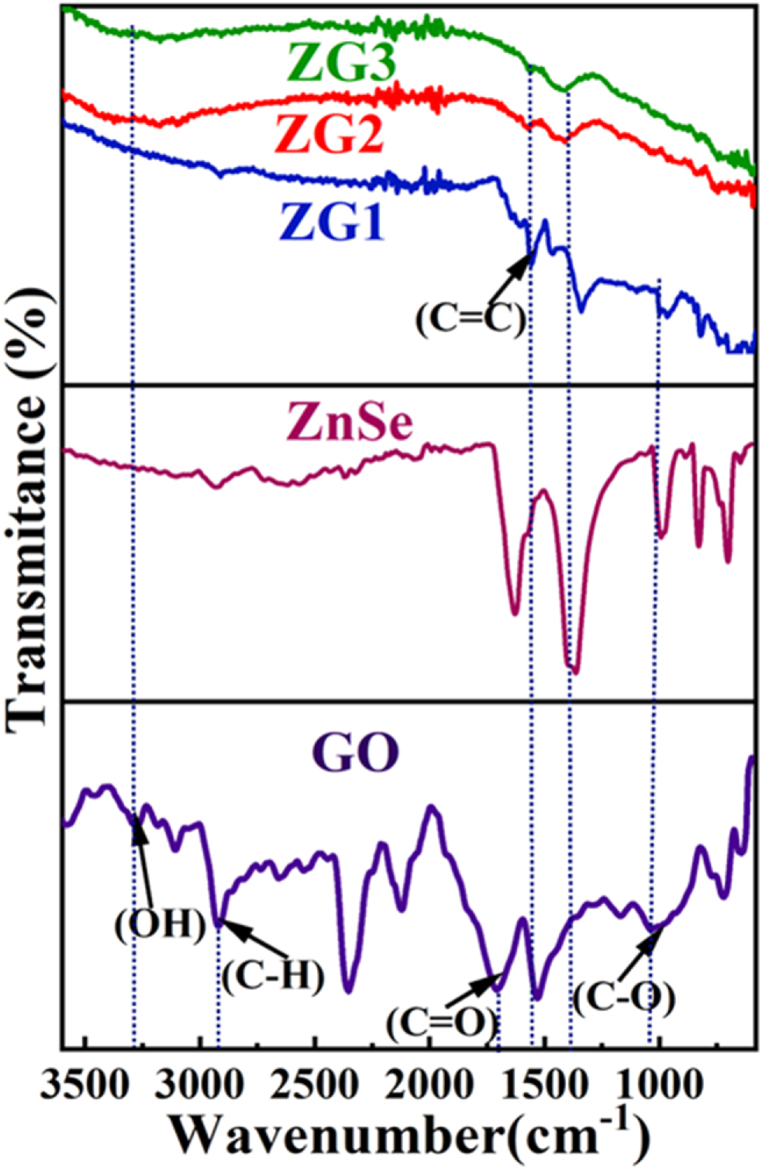
The comparative FTIR spectrum of ZG1, ZG2 and ZG3 nanocomposites along with GO and ZnSe.

The reduction of graphene oxide into rGO in ZnSe-XrGO nanocomposites were confirmed by FTIR spectra recorded at 500-4000 cm^−1^ wavenumber. The comparison of FTIR spectra of ZG1, ZG2 and ZG3 with GO and ZnSe are illustrated in Fig. 3. The peaks appeared at 3285 cm^−1^is due to O–H stretching vibration of hydrogenbond in water [30,45]. In the FTIR spectra of ZnSe-XrGO nanocomposites, all the absorption bandofcarbon oxygen bond (-C=O, C–O, and C–*O*–C)were completely gone. Only one peak at 1563 cm^−1^ is observed which corresponds to (C=C) confirming the successful reduction of graphene oxide in the nanocomposites [30,46,47].

### 3.3 Surficial morphology and elemental investigation

**Fig. 4:**
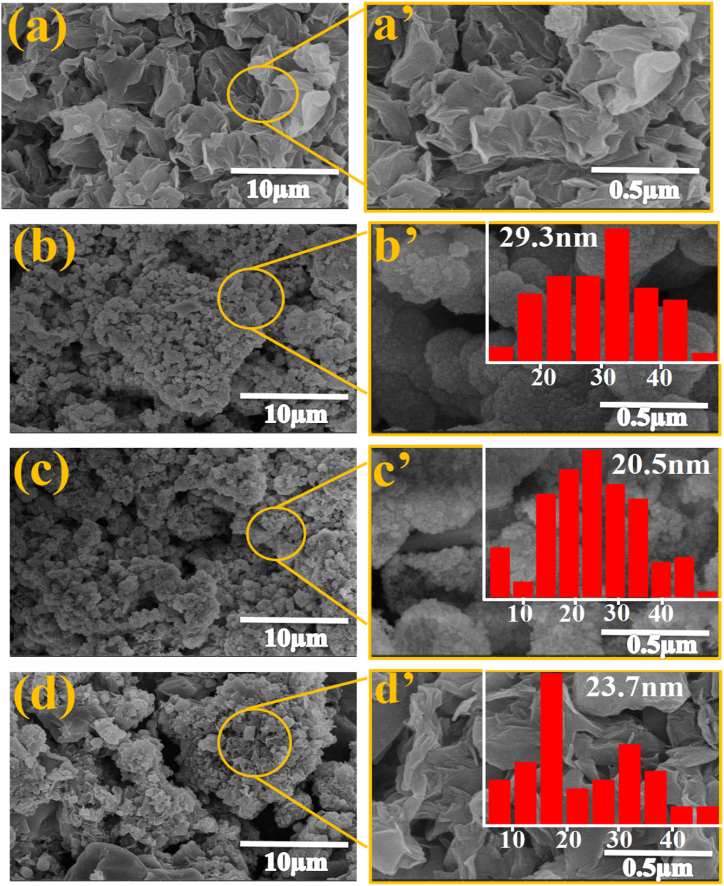
Scanning electron microscopic (SEM) images with different resolutions of nanocomposites (a) rGO (b) ZG1 (c) ZG2(d) ZG3. Insets in figure b’-d’ are particle size distribution of ZnSe NPs.

FE-SEM images of GO and ZnSe-XrGO nanocomposites are illustrated in Fig. 4a-d. It is clear from Fig. 4a that rGO has sheet like morphology with no extra impurity over the surface. The images in Fig. 4b-d corresponding to ZG1, ZG2 and ZG3, respectively, display mostly spherical ZnSe nanoparticles decorated on the sheets of rGO. The same figures are magnified for all the samples as Fig. 4(b’-d’). The morphology of the ZnSe was almost same as in Fig. 4(b’-c’) for ZG1 and ZG2 while graphene sheets are more prominent when the weight ratio of GO increases to 1.6 in ZG3. The particles of ZnSe are mostly agglomerated in the form of large clusters, which may be due to their chemical or physical characteristics [48,49]. The reduced graphene oxide sheets are responsible for many functions like improving the conductivity, increased binding sites, and defects enrichment of the ZnSe-XrGO photocatalyst [50]. The particle size distribution from SEM micrographs was estimated by using Image j and origin software and are shown in the insets of Fig. 4(b’-d’) for nanocomposites. Average particle size was calculated as 29.3 nm, 20.5 nm and 23.7 nm for ZG1, ZG2 and ZG3 respectively. It indicates that nanoparticles have size ranging between 15 and 20 nm and is good in agreement with the average particle size calculated by XRD data.

**Fig. 5:**
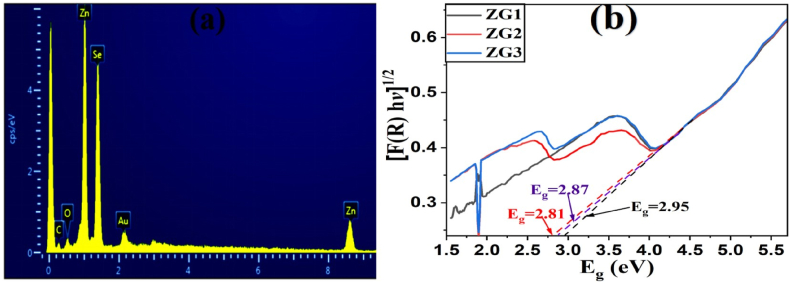
(a) Elemental composition in energy dispersive X-ray (EDX) spectrum of a representative ZG2nanocomposites (b) Kubelka-Munk reflectance plot of ZnSe-XrGO nanocomposites for estimation of band gap (E_g_).

The elemental composition of all the synthesized materials was assessed by the energy dispersive X-ray (EDX) analysis and the results of a representative ZG2 sample are given in Fig. 5a. It is obvious from the figure that C, O, Zn ad Se are present in the nanocomposite which further confirms the successful loading of ZnSe over rGO.

### 3.4 Optical properties and band gap

UV-VIS spectra were recorded for the measurements of optical properties of all the nanocomposites which were later converted into Kubelka-Munk function (Equation (6)) for estimating the band gap of synthesized materials [51]:

(6) $${F\left(R_{\infty }\right)h\nu )}^{1/2}=A\left(h\nu -E_{g}\right)$$

where, F(R) is function of reflectance, ν is frequency of light incident, h is Planck's constant, E_g_ is the optical bandgap and n = ½ for direct transitions. The kubelka-Munk transformation of reflectance spectra of nanocomposites have been shown in Fig. 5b. The ZG2 has direct band gap of 2.81eV which is least among all the other fabricated materials as 2.95eV for ZG1 &2.87eV for ZG3. The band gap of all the nanocomposites are higher than that of already reported ZnSe's band gap(i.e. 2.7eV) [30], which indicates that the recombination rate has been retarded for composite in comparison to individual nanostructure. The band edge positions were determined from the empirical estimation by Butler and Ginley(Butler & Ginley, 1978) and were found as E_CB_ = −0.74 eV and E_VB_ = 1.96 eV for ZnSe and E_CB_ = 1.105 eV and E_VB_ = 2.795 eV for rGO. The energy of highest energetic VB is of ZnSe in nanocomposite and the required threshold energy (E_i_) is 1.96eV for the transfer of electron from VB to CB. This energy corresponds to 632.6 nm light, that falls under visible region of solar spectrum. Overall the whole mechanism was predicted and is discussed later in this document. These results indicate that the nanocomposites have good visible light harvesting properties which ultimately lead to enhanced photocatalytic activity [52].

### 3.5 Photocatalytic degradation of textile dye (MV)

**Fig. 6:**
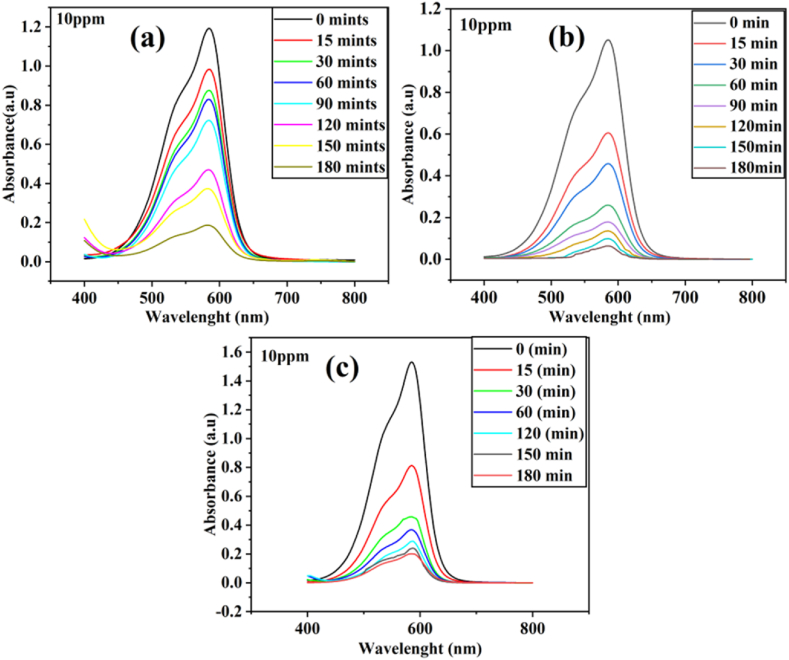
UV-VIS adsorption spectral changes of 10 ppm MV solution as a function of time over (a) ZG1 (b) ZG2 (c) ZG3 under Visible light irradiation.

**Fig. 7:**
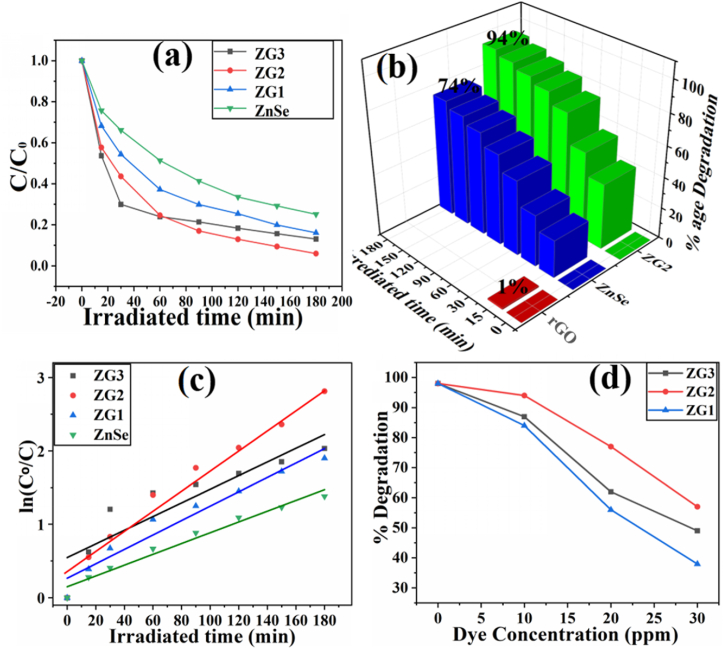
(a) Photocatalytic degradation trends (b) Photocatalytic degradation efficiency of ZG2 with GO and blank ZnSe nanoparticles(c) Plot of ln (C_0_/C) as a function of irradiated time(t) (d) Effect of concentrations of MV dye on the photodegradation efficiency over ZG1, ZG2, ZG3 nanocomposites.

The photocatalytic activities of ZnSe, GO & ZnSe-XrGO nanocomposites were investigated by photodegradation of MV dye under visible light irradiation. Temporal changes in concentration of the dye were monitored by examining the variations in maximal absorption in UV–visible spectra at 586nmthe UV–vis absorption spectra are presented in Fig. 6(a-c)for ZG1, ZG2 and ZG3 respectively. The absorption band's intensity is decreasing as a function of irradiation time, indicating photodegradation of MV, periodically [53]. The degradation profile of MV with time by the nanocomposites and ZnSe has been compared in Fig. 7a, b. The degradation of MV was 94 % with ZG2 but 87 %, 85 %, 74 % of MV for ZG1, ZG3 and ZnSe, respectively, under same experimental conditions. It is obvious that ZnSe-XrGO has comparatively high photocatalytic activities than ZnSe. The reason is that the reduced graphene oxide (rGO) in the nanocomposite has increased the mobility of photo-generated charges [35], retarded electron-hole pair recombination resulting in high electron density and provide high surface area for interaction with dye [27,54,55],

The pseudo-first ordered rate constant (k) was calculated by using equation (2). The plot of ln(C_0_/C) as a function of irradiated time over ZnSe-XrGO nanocomposites is given as Fig. 7c. The values of k (min^−1^)are obtained from the slope of equation (2) and the data is provided in Table 1. It is evident from the results that ZG2 has highest photodegradation efficiency (94 %) for MV dye and rate constant as 0.0167min^−1^ with R^2^ of 0.96 under visible light.

**Table 1:** Kinetic data for degradation of organic pollutant methyl violet by all synthesized samples.

Photocatalyst	k/min	R^2^	Degradation(%) ofMV	
GO	0.019	0.97	4 %	
ZnSe	0.0063	0.99	74 %	
ZG1	0.0060	0.92	84 %	
ZG2	0.0167	0.96	94 %	
ZG3	0.0079	0.80	88 %	

The plot of %age degradation with respect to initial dye concentration of MV, using ZG1, ZG2 and ZG3 has been shown in Fig. 7d. All the photocatalysts showed the same pattern of degradation in all cases. It was observed that high degradation efficiency at low initial concentration of dye (C_o_ = 10 ppm) is due to high adsorption of most of the dye molecules on the surface of the photocatalyst and instant degradation under same conditions. As the initial concentration of MV in solution increases further from 10 ppm, the percentage degradation decreases, which may be attributed to the lesser light penetration and increased competition for dye molecules to approach on the available active sites of the photocatalyst for adsorption. Furthermore, large number of dye molecules adsorb on the surface of ZnSe-XrGO nanocomposites, but will not be degraded instantly, as the catalyst amount and intensity of light are still constant [52].

Conclusively, as the weight ratio of GO to ZnSe increases from 0.6 (ZG1) to1 (ZG2), the degradation of dye also increases from 84 % to 94 %. Further increment in weight ratio of GO:ZnSe (up to 1.6) has decreased the degradation efficiency of ZG3 to 87 %. The increased activity of ZG2 is attributed to higher crystallinity (57 %) and least particle size (20.5 nm) among all the different samples as estimated from XRD and SEM, respectively. The only GO is not really active for MV degradation but when combined with ZnSe in 1:1 it has majestic effect over the photocatalytic activity for MV degradation [52].

The specialty of our ZnSe-rGo nanocomposite is it's visible light active and highly cost-effective. The comparison of synthesized photocatalyst with already reported photocatalyst in literature is given in Table 2. Which indicates that ZnSe-rGO have high degradation efficiency under visible light. Other photocatalysts have mostly used UV or highly intense sunlight.

**Table 2:** Comparison of photocatalytic performance of ZnSe/rGO for MV degradation with already reported photocatalysts. (where **CV** = Crystal Violet, **MV** = Methyl violet, **MB** = Methylene Blue).

Photocatalyst	Dye	Light source	Time (min)	Degradation (%)	Reference
CuCo_2_O_4_	MV	Sunlight	60	93	[56]
GO/NiS	CV	Sunlight	80	91.01	[57]
SnO_2_	MV	Sunlight	120	80	[58]
Mg-doped ZrO_2_	MV	UV	70	92	[59]
Co(II) based coordinate polymer	MV	UV	50	86.1	[60]
NiO/Cr_2_O_3_	CV	Sun light	180	88.47	[61]
ZnS–ZnO NC	CV	UV	180	88.36	[62]
ZnS QD	MV	UV	120	91.4	[63]
MgFe_2_O_4_/ZnO/GO	CV	Visible light	120	80	[64]
ZnS-rGO	MB	Sunlight	120	73.73	[65]
Zn(II)- MOFs	MV	UV	40	55.95	[66]
ZnO/NiFe_2_O_4_@rGO	CV	Sunlight	140	80.05	[67]
ZnO	MV	Sunlight	90	81	[68]
ZnSe	MV	Visible light	180	74	This work
ZnSe/rGO	MV	Visible light	180	94	This work

### 3.6 Predicted mechanism of photodegradation of MV

**Scheme 1:**
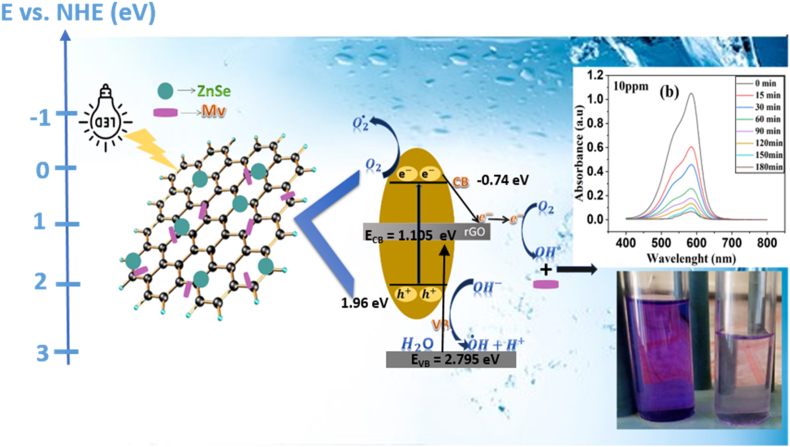
Representation of degradation of MV by ZnSe-rGO photocatalyst under visible light irradiation.

**Scheme 2:**
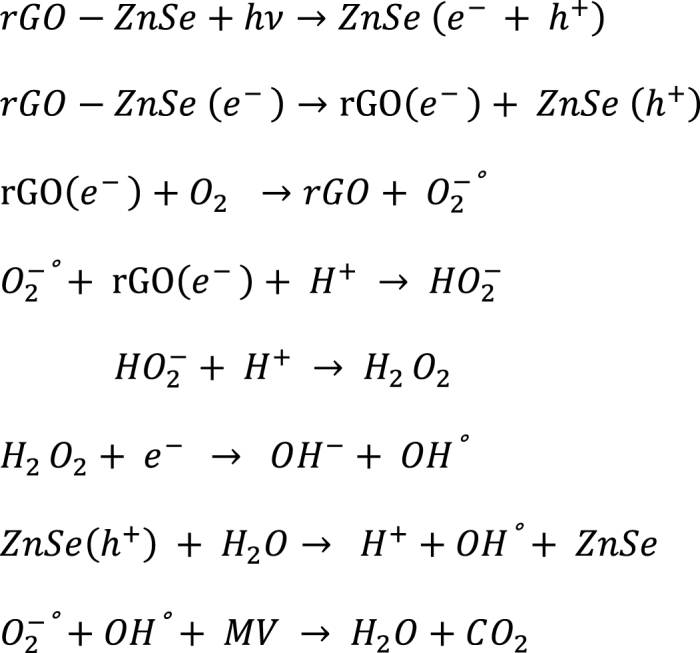
Schematic pathway for active oxygen radical generation and MV dye degradation by ZnSe:XrGO nanocomposite.

The predicted mechanism of photodegradation of methyl violet (MV) is presented in Scheme 1. When ZnSe-XrGO nanocomposite was exposed to visible-light, the electrons (e^−^) were excited from valence band (VB) to conduction band (CB) of ZnSe, leaving behind holes (h^+^) in its VB. Due to favorable energy levels of CB of ZnSe and graphene's chemical potential, electrons get transferred to graphene sheets causing more electrons from VB to excite in the CB of ZnSe. In this way, the graphene sheets not only facilitate the adsorption of dye molecules over the nanocomposite surface but also aids in charge separation. The well-separated photoinduced electrons and holes initiated a chain reaction that produces active oxygen based radicals, illustrated as Scheme 1, Scheme 2. The existence of hydroxyl group (a key factor) presents a high affinity for positively charged nitrogen in MV molecules, facilitating adsorption via electrostatic interactions, and break the long chain conjugation of dye converting it into simpler, non-toxic products (H_2_O, CO_2_) [30,69].

This whole processes is represented in Scheme 2. [30]:

$$rGO-ZnSe+h\nu \to ZnSe\left(e^{-}+h^{+}\right)$$

$$rGO-ZnSe\left(e^{-}\right)\to \text{rGO}\left(e^{-}\right)+ZnSe\left(h^{+}\right)$$

$$\text{rGO}\left(e^{-}\right)+O_{2}\to rGO+O_{2}^{-{}^{\circ}}$$

$$O_{2}^{-{}^{\circ}}+\text{rGO}\left(e^{-}\right)+H^{+}\to {HO}_{2}^{-}$$

$${HO}_{2}^{-}+H^{+}\to H_{2}O_{2}$$

$$H_{2}O_{2}+e^{-}\to OH^{-}+OH{}^{\circ}$$

$$ZnSe\left(h^{+}\right)+H_{2}O\to H^{+}+OH{}^{\circ}+ZnSe$$

$$O_{2}^{-{}^{\circ}}+OH{}^{\circ}+MV\to H_{2}O+CO_{2}$$

### 3.7 Effect of dye contaminated water on germination and growth parameters of maize

**Fig. 8:**
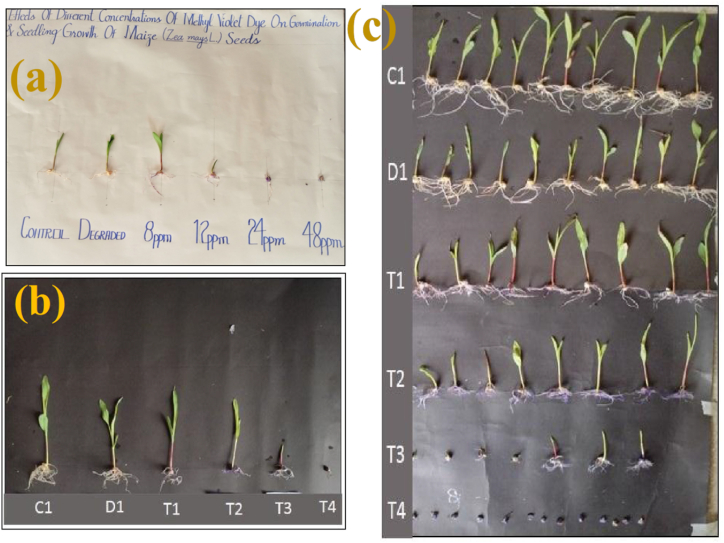
Comparison of maize seedling growth at different concentrations of dye at (a) 7th day (b) 10th day of seed sowing (c) Comprehensive representation of seedling growth and germination in maize plants.

Preliminary evidence for negative or positive effect of MV dye and after its photodegradation on plant growth can be collected by evaluating %age germination of the seed and growth of plants at the early growth stages. Effect of MV polluted water on the seed germination of maize plant was monitored by making different treatment levels for seedling growth. Varying MV dye concentration were 0.0 ppm solution (control, C1), degraded MV dye solution (D1), 8.0 ppm dye solution (T1), 12 ppm dye solution (T2), 24 ppm dye solution (T3) and 48 ppm dye solution (T4). Seed germination and seedling growth of maize were assessed after 7 and 10 days of sowing (Fig. 8a–c). A comprehensive display of replicates of each treatment after 10 days of sowing is given in Fig. 8c. The results show that moderate to higher levels of MV i.e., 12–48 ppm caused a substantial decrease in percent seed germination and seedling growth after 7 and 10 days of sowing. Photodegraded MV dye (D1) and low concentration of MV dye (8 ppm contaminant level; T1) almost did not affect the percent seed germination and seedling growth of maize after 7 days of sowing. However, after 10 days of sowing, both these treatments (D1, T1) had slightly negative effect on growth of maize seedlings. This variable retarding effect of MV at lower concentration or at the higher concentration on seedling growth of maize might have been due to variable blockage of water uptake by virtue of dye adsorbed on the roots.

**Fig. 9:**
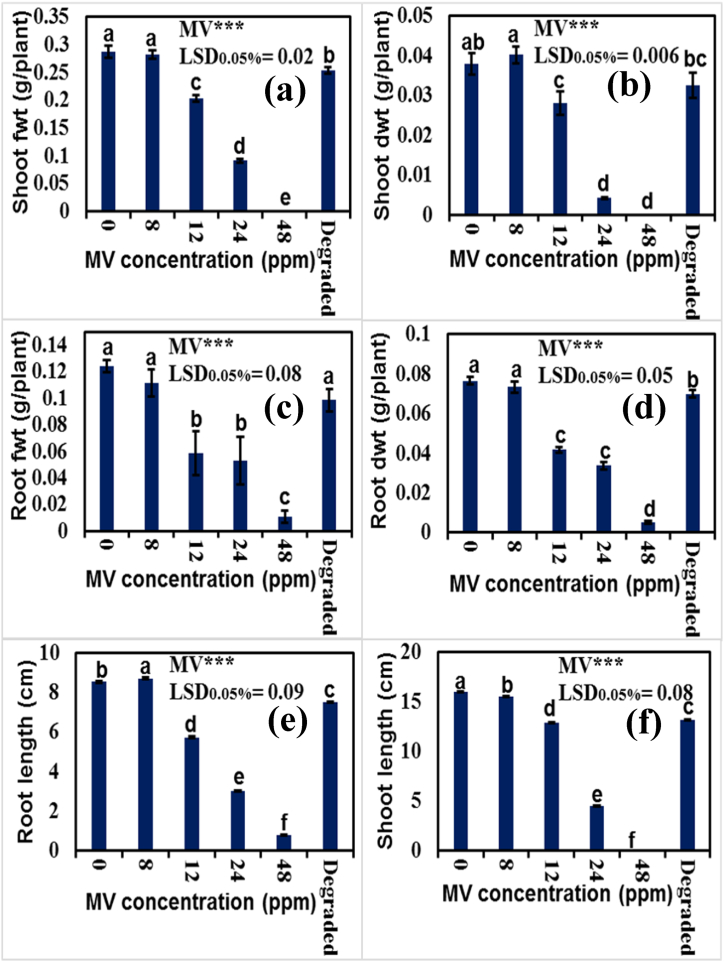
Comparison of trends of (a, b) shoot fresh and dried weight (c, d) root fresh and dried weight (e, f) length of root and shoot of maize plants at different dye contamination levels are shown respectively.

Increasing concentration of MV dye in the growth medium caused the reduction in shoot and root lengths (Fig. 9e and f), particularly at the highest MV dose (48 ppm) where maize seeds did not germinate even (Fig. 9a). However, treatment with photodegraded MV dye (D1 treatment) has the least reducing effect on growth of maize seedlings. Similarly, fresh and dry weights of shoots and roots (Fig. 9a–d) consistently decreased with consistent increase in MV dye concentration in rooting medium except at 8 ppm MV dose, where its negative effect was least on these growth attributes of maize. In addition, application of photodegraded MV dye (D1) had the least negative effect on these growth attributes of maize plants. These results are analogous to some already published articles in which it has been documented that 8 ppm textile azo dyes improved the seedling growth initially, and then had some negative effects on seedling growth upon exposure for longer period in soybean, chickpea, mung bean and mash bean etc. [[70], [71], [72]].

**Fig. 10:**
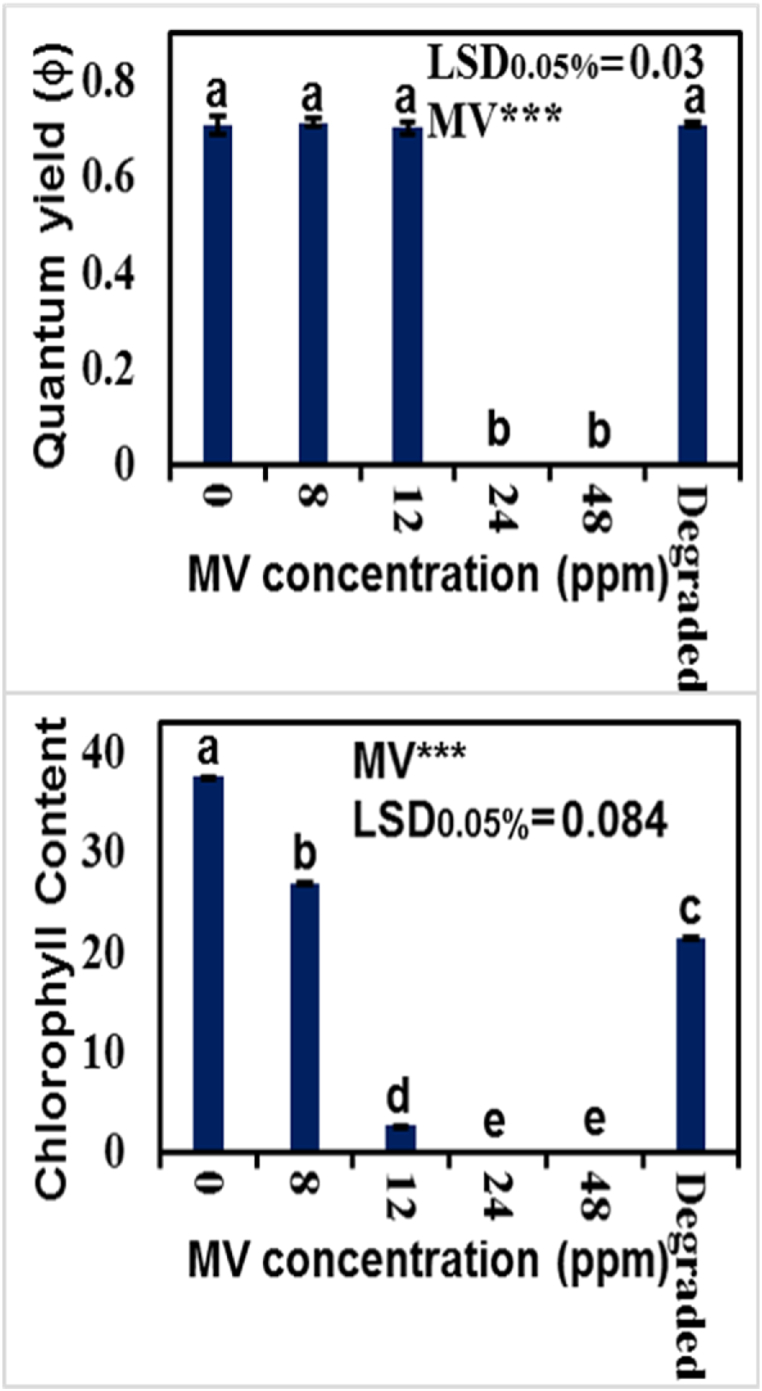
Comparison of (a) Quantum yield of PSII (b) Chlorophyll contents of maize plants at different dye contamination levels. Means were compared with LSD at 5 % probability.

Plant growth is mainly translated by plant photosynthetic capacity. Amount of photosynthetic pigments, and activity of photosystem II (PSII) are key factors that determines the plant'stability to capture the solar energy and convert it in biochemical energy for CO_2_ fixation. The quantum yield of PSII in maize plants remained almost unchanged in all maize plants grown under normal conditions or under varying concentration of MV dye levels, except 24 and 48 ppm level where seeds were either not germinated or poorly germinated. Although maize seedlings treated with degraded MV dye exhibited slight inhibition in growth, there was no change in quantum yield of PSII (Fig. 10a). The chlorophyll contents were reduced in the leaves of maize plants treated with 8 and 12 ppm MV dye. Application of degraded MV dye reduced chlorophyll content in the leaves of maize plants, however, amount of chlorophyll content was greater in photodegraded MV dye treated plants than those of 12 ppm MV de treated maize plants (Fig. 10b). These results indicated that MV dye either inhibited chlorophyll biosynthesis or promoted the chlorophyll degradation in maize plants. Though photodegraded MV dye had the least negative effect on growth of maize plants, it reduced the chlorophyll content. However, the mechanism of MV dye in regulating chlorophyll content in plants is not known. Conclusively, the germination and seedling growth of maize plant was negligible when supplied with 24–48 ppm solution while it was significantly improved by supplying it with the water after dye degradation by using ZnSe-XrGO catalyst.

**Fig. 11:**
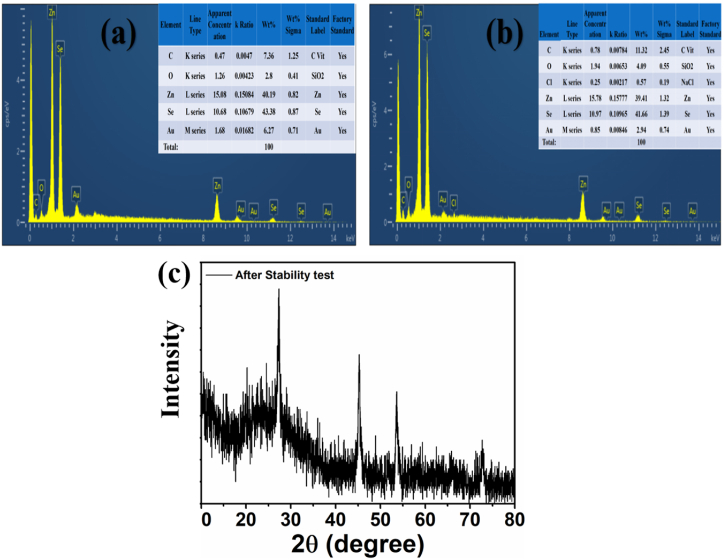
(a–b) EDX of ZG2 sample before and after stability test, (c) XRD pattern after stability test.

In order to ensure the material's stability, the ZG2 sample was re-tested with EDX and XRD after the degradation of MV and the results are presented in Fig. 11(a–c). It is obvious from the figure that the crystal structure is still intact (Fig. 11c), and also the elemental composition supports the same where Zn, Se, C, and O are the major constituents of ZG2 before and after photocatalytic reaction. In short, an efficient, cost effective, visible light active photocatalyst is presented in this work that effectively degrades MV contaminated solution and make the water as harmless as the pure one, for real life samples.

## 4 Conclusion

The MV dye contaminations in water may affect the aquatic and terrestrial life by retarding plant's growth, reducing the cell activities, inducing various diseases and deficiencies due to accumulation in the cells of living organisms. The current research emphasizes the removal of MV dye from water by using ZnSe-XrGO nanocomposites, fabricated in different weight ratios (ZnSe:rGO) via one-step hydrothermal method. The results indicate that photodegradation efficiency of the ZnSe-XrGO nanocomposites for MV, was increased with increasing the weight ratio of GO from (0.6–1), which may be due to reduction in recombination rate, high crystallinity and enhancement of charge separation by GO. The highest efficiency was achieved by ZG2(i.e. 94 % degradation in 3 h), when ZnSe:rGO was 1:1. The optimized sample followed pseudo 1st order behavior with rate constant of 0.0167min^−1^for MV photodegradation. Furthermore, growth of maize plant is reduced at moderate to higher contents of MV (12–48 ppm). However, photodegradation of MV dye make it least harmful for plant growth as it slightly reduced the chlorophyll content and growth of maize seedling growth but it did not affect the quantum yield of PSII. Overall, a simple, cost effective and versatile photocatalyst based on ZnSe-rGO was prepared, that has its direct applications for improving the quality of water for a sustainable life.

## Data availability statement

Data included in article/supp. material/referenced in article. No other data will be available publicly.

## CRediT authorship contribution statement

**Mishal Zahra:** Writing – original draft, Methodology, Investigation, Conceptualization. **Ghazala Yasmeen:** Writing – review & editing, Project administration, Conceptualization. **Faryal Aftab:** Writing – review & editing, Software, Investigation, Conceptualization. **Habib-ur-Rehman Athar:** Writing – review & editing, Investigation, Data curation. **Aisha Saleem:** Validation, Software, Data curation. **Sarah Ambreen:** Investigation, Formal analysis, Conceptualization. **Muhammad Aslam Malana:** Writing – review & editing, Project administration, Conceptualization.

## Declaration of competing interest

The authors declare that they have no known competing financial interests or personal relationships that could have appeared to influence the work reported in this paper.
